# State Veterinarians

**Published:** 1885-10

**Authors:** 


### STATE VETERINARIANS.

It is a sign of the advancement of our profession in public appreciation that several States have lately made the position of State Veterinarian one of the regular appointments of the Governor or their Legislatures. It is singular that nearly all these appointments have been made in western States, and to their credit also, that the majority of them have been conferred on regularly graduated men.

Professor Liautard of the American Veterinary College of New York City, could certainly ask no better endorsement from the public, of his self-sacrificing endeavors to elevate his and our profession, than the fact, that most of the men thus selected have been graduates of the American Veterinary College.

If these positions can be assured to veterinarians as permanent, and free from political influence so long as the occupant does his duty, the governments of the different States can be assured of obtaining the very best among the young men in the profession to fill them. Otherwise they will have to take up with what they can get, for no man can afford to give up the competency which he can gain in practice for any temporary official position.

The Governor of Nebraska, in selecting Dr. Julius Gerth, jr., as State Veterinarian, is to be congratulated, and we can assure him that his selection will not fail him.

The writer probably knows Dr. Gerth better than any other person in the country. His career has been one of remarkable success, and it is well that we place before our colleagues the reasons therefor.

With many disadvantages, the greatest of which was his youth, with an education in no way superior to that of his colleagues in the profession, he has gradually worked his way into a position of respect and influence in his native State, New

Jersey. The reason of this success is to be sought in the fact that at school he became instilled with the true principles of veterinary science, and came to a knowledge of the true relation of the veterinarian to the State, as an officer of the public service, and an active agent in the prevention of human disease, as well as the contagio-infectious diseases which play such an essential role in national economy.

From the day he first entered practice, Dr. Gerth has made these principles the guiding star of his life. He has done more than any one else in his State to impress upon the authorities the fact that they owe a certain amount of deference to the educated veterinarian over the uneducated empiric, which fact has borne fruit in the endorsements of the State Board of Health, and that of the city of Newark, in their actions and appointments.

Having thus studied these conditions, Dr. Gerth has shown that his abilities lie mostly in the direction of organizing and directing a veterinary sanitary police system. He is also remarkably free from the curse which has thus far disgraced most of the veterinary officials in this country—which has been the fear of appointing men of real ability to act under them. We know that the State Veterinarian of Nebraska has none of this smallness in his nature, and that he realizes that the better qualified his assistants are the better will be the work done in his department, Our profession may be assured that he will work for its elevation even more than he will for his own personal gain. Success to him, and every other State Veterinarian is the wish of this journal.

